# Publisher Correction: Multi-experiment nonlinear mixed effect modeling of single-cell translation kinetics after transfection

**DOI:** 10.1038/s41540-019-0083-6

**Published:** 2019-03-07

**Authors:** Fabian Fröhlich, Anita Reiser, Laura Fink, Daniel Woschée, Thomas Ligon, Fabian Joachim Theis, Joachim Oskar Rädler, Jan Hasenauer

**Affiliations:** 1 Institute of Computational Biology, Helmholtz Zentrum München, 85764 Neuherberg, Germany; 2 Center for Mathematics, Technische Universität München, 85748 Garching, Germany; 3 Faculty of Physics and Center for NanoScience, Ludwig-Maximilians-Universität, 80539 München, Germany; 4 Faculty of Mathematics and Natural Sciences, Rheinische Friedrich-Wilhelms-Universität Bonn, 53115 Bonn, Germany

**Correction to:** *npj Systems Biology and Applications* 10.1038/s41540-018-0079-7, Published online: 10 December 2018

The original version of this Article had an incorrect Article number of 1, an incorrect Volume of 5 and an incorrect Publication year of 2019. These errors have now been corrected in the PDF and HTML versions of the Article.

