# Supplementary material for: Multi-experiment nonlinear mixed effect modeling of single-cell translation kinetics after transfection
Source: NPJ Syst Biol Appl. 2018 Dec 10;4:42. doi: 10.1038/s41540-018-0079-7 (PMC6288153; doi:10.1038/s41540-018-0079-7)
Supplement: Supplementary file 1 — Supplementare Information [file 41540_2018_79_MOESM1_ESM.pdf]

# Supplementary Information

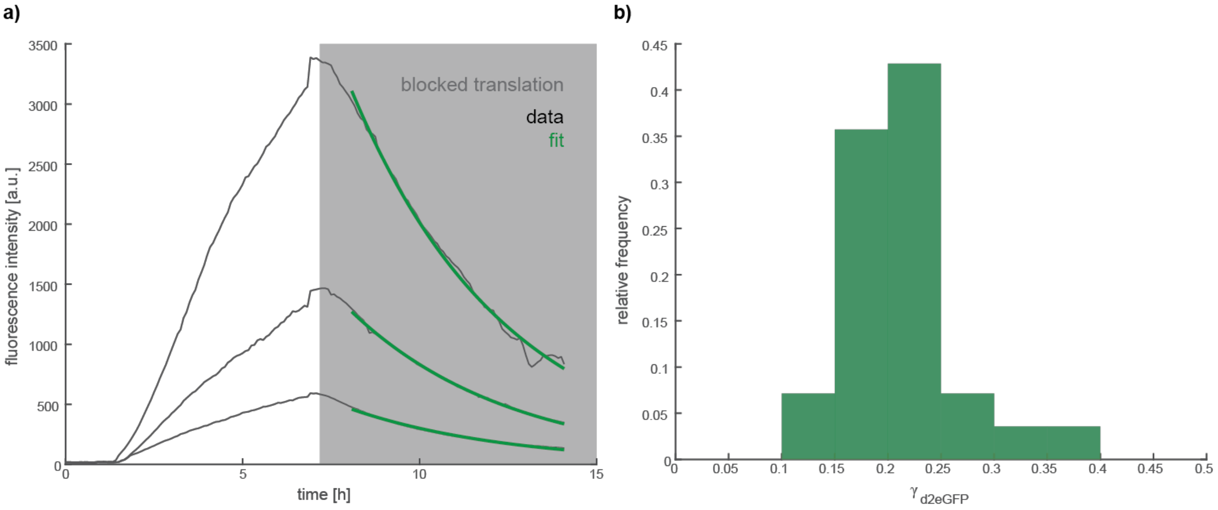


**Figure S1: Validation of first order degradation kinetics. a)** Single-cell d2eGFP expression measured by integration over the fluorescence intensity in a translation block experiment. The translation inhibitor cycloheximide was added 7 hours post transfection. Three single cell traces (grey lines) are shown with the corresponding fits of exponential decays (green lines) to the time from 8 to 14 hours post transfection. **b)** Distribution of the decay rates for d2eGFP obtained by fitting the single cells individually.

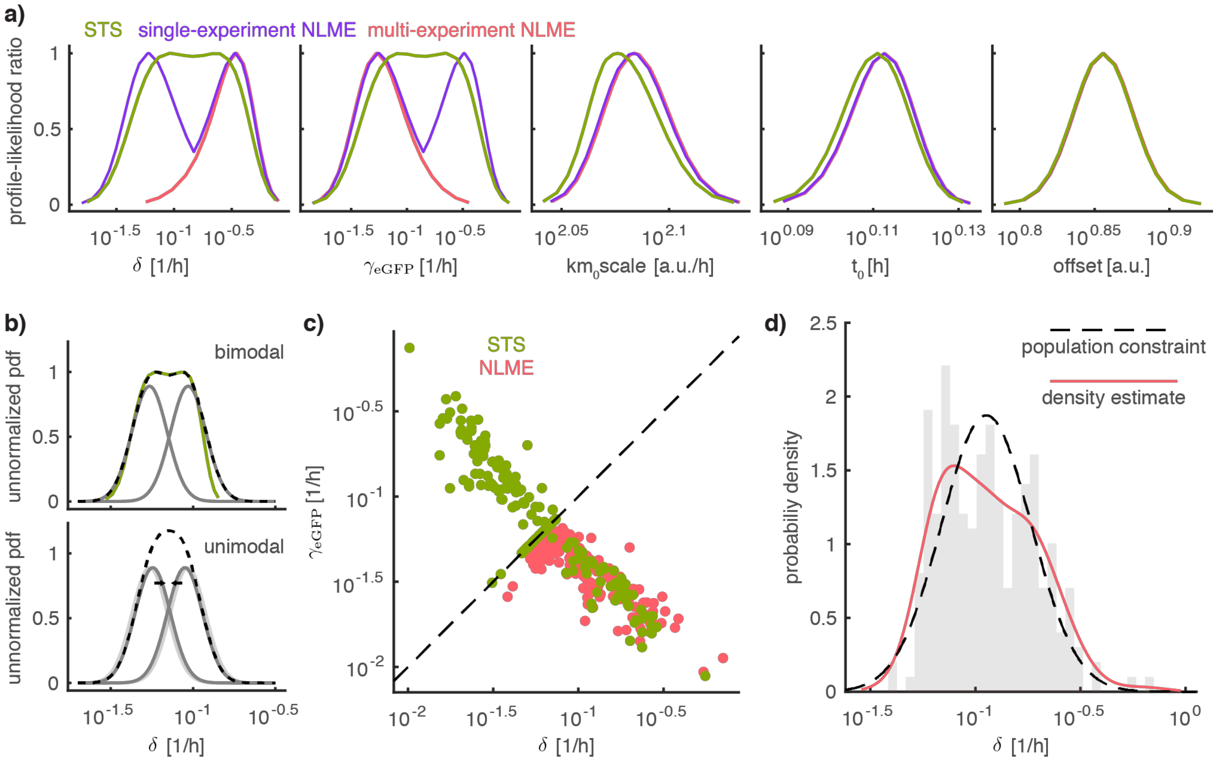


**Figure S2 Analysis of single-cell parameters for the STS and the NLME approach for the eGFP dataset for a single cell. a)** Profile-Likelihood ratios of single-cell parameters for the STS approach ($\theta_i$, green), the single-experiment NLME approach ($\varphi_i$, purple) and for the multi-experiment NLME approach ($\varphi_i$, red). For the NLME approach profiles, the estimated population parameters $\beta$ and $D$ were fixed during profile calculation. For the single-experiment NLME approach profiles from local minima in the parameters $\beta$ and $D$ were combined. Color indicates the employed approach. **b)** Sketch explaining the emergence of unimodal and bimodal profile shapes. The dashed black line is the superposition of the two normal densities in grey. The computed profile-likelihood is shown as green line. **c)** Comparison of estimated single-cell parameters for the STS ($\theta_i$, green) and NLME ($\theta_i$, green) approach. **d)** Comparison of NLME single-cell parameters histogram (bars) and kernel density estimate (red line) with the population parameter distribution (black dashed line).

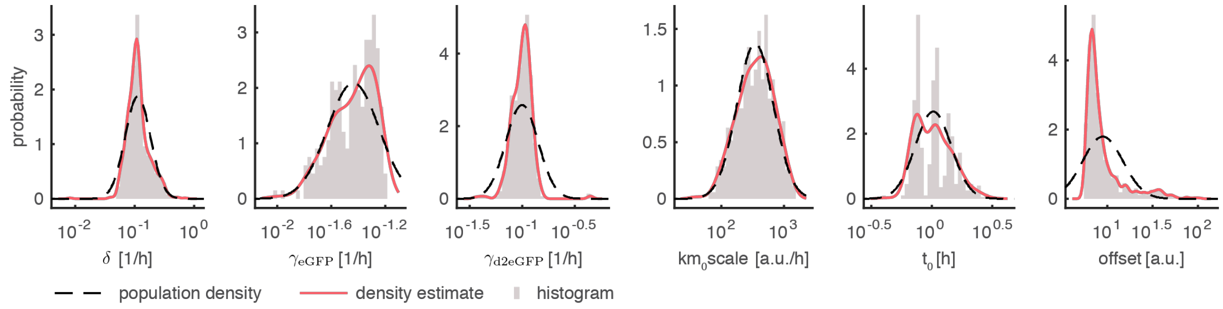


**Figure S3 Comparison of distributions of estimated single-cell parameters ($\varphi_i$) and estimated population parameter distributions ($\beta, D$).** NLME single-cell parameters histogram (bars) and kernel density estimate (red line) with the population constraint (black dashed line).

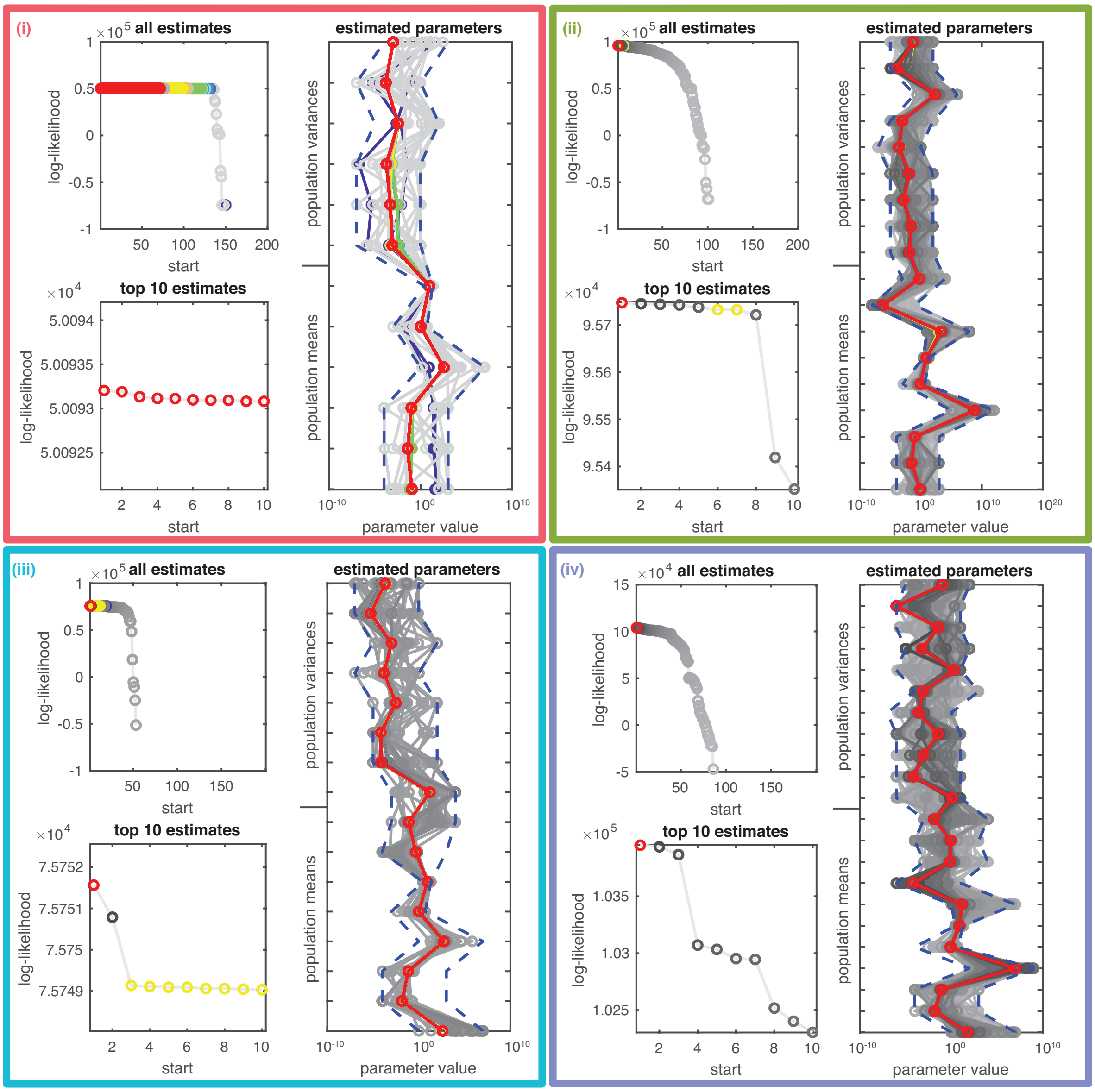


**Figure S4 Analysis of reproducibility of optimization results for the NLME approach.** Results for each considered model are shown in individual subplots, which are marked by roman numerals and border color. In each subplot, the left two plots show all/top10 log-likelihood values across all optimization runs. Highest objective function value is always colored in red. For all other optimization runs, coloring is only applied if multiple optimization runs yielded objective function values within 0.01 of each other, otherwise a grey color indicates the rank in log-likelihood value. The group of best estimates is always shown in red. The same coloring is applied to the estimated parameter values on the right. Parameter boundaries are indicated as dashed blue lines. For most models, colored lines and dark grey lines are barely visible due to overlay of the optimal parameter in red. Crashed optimization runs are not shown.

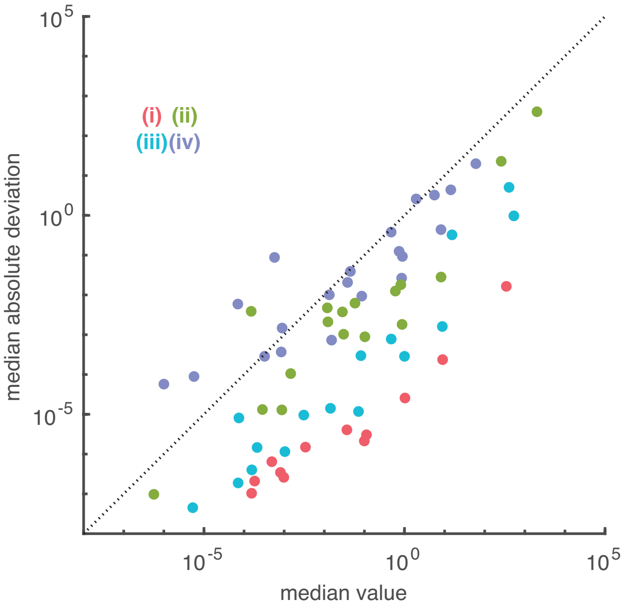


**Figure S5 Analysis of reproducibility of parameter estimates for the NLME approach.** Each dot corresponds to a single parameter. Median and median absolute for the endpoints of the five local optimizer runs which reached the highest log-likelihood values are depicted.

**Table S1 Mathematical Formulation and Transformation of Models (i) - (iv).** Molecule abundances are abbreviated for mRNA (m), protein (p), ribosome (r), enzyme (e), ribosome-mRNA complex (rm) and enzyme-mRNA complex (em).

| Model | ODE | | transformed ODE | | Transformation |
|---|---|---|---|---|---|
| model (i) | $\frac{dm'}{dt} = -\delta \cdot m'$ | $m'(t_0) = m_0$ | $\frac{dm}{dt} = -\delta m$ | $m(t_0) = 1$ | $m = \frac{m'}{m_0}$ |
| | $\frac{dp'}{dt} = k \cdot m - \gamma \cdot p'$ | $p'(0) = 0$ | $\frac{dp}{dt} = km_0\text{scale} \cdot m - \gamma \cdot p$ | $p(0) = 0$ | $p = \text{scale} \cdot p'$ |
| | $y = \log(\text{scale} \cdot p' + \text{offset})$ | | $y = \log(p + \text{offset})$ | | |
| | parameters : $\delta, m_0, k, \gamma, \text{scale}, \text{offset}$ | | $\delta, km_0\text{scale}, \gamma, \text{offset}$ | | |
| model (ii) | $\frac{dm'}{dt} = -\delta \cdot m' - k_1 \cdot m' \cdot r' + k_2 \cdot rm'$ | $m'(t_0) = m_0$ | $\frac{dm}{dt} = -\delta \cdot m - k_1 m_0 \cdot m \cdot r + k_2 \cdot (\frac{r_0}{m_0} - r)$ | $m(t_0) = 1$ | $m = \frac{m'}{m_0}$ |
| | $\frac{dr'}{dt} = k_2 \cdot rm' - k_1 \cdot m' \cdot r'$ | $r'(0) = r_0$ | $\frac{dr}{dt} = k_2 \cdot (\frac{r_0}{m_0} - r) - k_1 m_0 \cdot m \cdot r$ | $r(0) = \frac{r_0}{m_0}$ | $r = \frac{r'}{m_0}$ |
| | $\frac{drm'}{dt} = k_1 \cdot r' \cdot m' - k_2 rm'$ | $rm'(0) = 0$ | | | $r_0 - r = rm'$ |
| | $\frac{dp'}{dt} = k_2 \cdot rm' - \gamma \cdot p'$ | $p'(0) = 0$ | $\frac{dp}{dt} = k_2 m_0\text{scale} \cdot (\frac{r_0}{m_0} - r) - \gamma \cdot p$ | $p(0) = 0$ | $p = \text{scale} \cdot p'$ |
| | $y = \log(\text{scale} \cdot p' + \text{offset})$ | | $y = \log(p + \text{offset})$ | | |
| | parameters : $\delta, m_0, k_1, k_2, r_0, \gamma, \text{scale}, \text{offset}$ | | $\delta, k_2 m_0\text{scale}, k_2, k_1 m_0, \frac{r_0}{m_0}, \gamma, \text{offset}$ | | |
| model (iii) | $\frac{dm'}{dt} = -\delta_1 \cdot m' \cdot e'$ | $m'(t_0) = m_0$ | $\frac{dm}{dt} = -\delta_1 m_0 \cdot m \cdot e$ | $m(t_0) = 1$ | $m = \frac{m'}{m_0}$ |
| | $\frac{de'}{dt} = \delta_1 \cdot m' \cdot e' - \delta_2 \cdot de'$ | $e'(0) = e_0$ | $\frac{de}{dt} = \delta_1 m_0 \cdot m \cdot e - \delta_2 \cdot (\frac{e_0}{m_0} - e)$ | $e(0) = \frac{e_0}{m_0}$ | $e = \frac{e'}{m_0}$ |
| | $\frac{dem'}{dt} = \delta_2 \cdot de' - \delta_1 \cdot m' \cdot e'$ | $em'(0) = 0$ | | | $e_0 - e = em'$ |
| | $\frac{dp'}{dt} = k \cdot m - \gamma \cdot p'$ | $p'(0) = 0$ | $\frac{dp}{dt} = km_0\text{scale} \cdot m - \gamma \cdot p$ | $p(0) = 0$ | $p = \text{scale} \cdot p'$ |
| | $y = \log(\text{scale} \cdot p' + \text{offset})$ | | $y = \log(p + \text{offset})$ | | |
| | parameters : $\delta_1, \delta_2, e_0, m_0, k, \gamma, \text{scale}, \text{offset}$ | | $\delta_1 m_0, \delta_2, \frac{e_0}{m_0}, km_0\text{scale}, \gamma, \text{offset}$ | | |
| model (iv) | $\frac{dm'}{dt} = -\delta_1 \cdot m' \cdot e' - k_1 \cdot m' \cdot r' + k_2 \cdot rm'$ | $m'(t_0) = m_0$ | $\frac{dm}{dt} = -\delta_1 m_0 \cdot m \cdot e - k_1 m_0 \cdot m \cdot r + k_2 \cdot (\frac{r_0}{m_0} - r)$ | $m(t_0) = 1$ | $m = \frac{m'}{m_0}$ |
| | $\frac{de'}{dt} = \delta_1 \cdot m' \cdot e' - \delta_2 \cdot de'$ | $e'(0) = e_0$ | $\frac{de}{dt} = \delta_1 m_0 \cdot m \cdot e - \delta_2 \cdot (\frac{e_0}{m_0} - e)$ | $e(0) = \frac{e_0}{m_0}$ | $e = \frac{e'}{m_0}$ |
| | $\frac{dem'}{dt} = \delta_2 \cdot de' - \delta_1 \cdot m' \cdot e'$ | $em'(0) = 0$ | | | $e_0 - e = em'$ |
| | $\frac{dr'}{dt} = k_2 \cdot rm' - k_1 \cdot m' \cdot r'$ | $r'(0) = r_0$ | $\frac{dr}{dt} = k_2 \cdot (\frac{r_0}{m_0} - r) - k_1 m_0 \cdot m \cdot r$ | $r(0) = \frac{r_0}{m_0}$ | $r = \frac{r'}{m_0}$ |
| | $\frac{drm'}{dt} = k_1 \cdot r' \cdot m' - k_2 rm'$ | $rm'(0) = 0$ | | | $r_0 - r = rm'$ |
| | $\frac{dp'}{dt} = k_2 \cdot rm' - \gamma \cdot p'$ | $p'(0) = 0$ | $\frac{dp}{dt} = k_2 m_0\text{scale} \cdot (\frac{r_0}{m_0} - r) - \gamma \cdot p$ | $p(0) = 0$ | $p = \text{scale} \cdot p'$ |
| | $y = \log(\text{scale} \cdot p' + \text{offset})$ | | $y = \log(p + \text{offset})$ | | |
| | parameters : $\delta_1, \delta_2, e_0, m_0, k_1, k_2, r_0, \gamma, \text{scale}, \text{offset}$ | | $\delta_1 m_0, \delta_2, \frac{e_0}{m_0}, k_2 m_0\text{scale}, k_2, k_1 m_0, \frac{r_0}{m_0}, \gamma, \text{offset}$ | | |

**Table S2 Comparison of estimated population mean values to literature values for the NLME approach with model (ii).**

| Parameter | Formula | Estimated value | Literature value | Reference |
|---|---|---|---|---|
| mean mRNA (all genes) half-life | $\frac{ln(2)}{\delta}$ | 0.8h | 1-30h | Schwanhäusser et al. *Nature* 473, 337, 2011 |
| mean eGFP half-life | $\frac{ln(2)}{\gamma_{eGFP}}$ | 22.8h | ~26h | Corish & Tyler-Smith. Protein Engineering 12, 1035-1040, 1999 |
| mean d2eGFP half-life | $\frac{ln(2)}{\gamma_{d2eGFP}}$ | 6.6h | ~5.5h | Corish & Tyler-Smith. Protein Engineering 12, 1035-1040, 1999 |
